# Study on hematological alterations induced by amphistomosis in buffaloes

**DOI:** 10.14202/vetworld.2015.417-420

**Published:** 2015-03-28

**Authors:** Vandip. D. Chauhan, P. V. Patel, Jigar J. Hasnani, Suchit S. Pandya, Sunanda Pandey, Dhaval V. Pansuriya, Vijayata Choudhary

**Affiliations:** 1 Department of Veterinary Parasitology, College of Veterinary Science and Animal Husbandry, Anand Agricultural University, Anand, Gujarat, India; 2 Department of Veterinary Pathology, College of Veterinary Science and Animal Husbandry, Anand Agricultural University, Anand, Gujarat, India; 3 Department of Veterinary Surgery and Radiology, College of Veterinary Science and Animal Husbandry, Navsari Agricultural University, Navsari, Gujarat, India

**Keywords:** amphistomosis, hematology, eosinophilia, anemia, neutrophilia

## Abstract

**Aim:**

The study was undertaken to compare the alterations in the hematological parameters in buffaloes suffering from Amphistomosis with normal buffaloes and to correlate it with the subclinical infection that is hard to diagnose.

**Materials and Methods:**

Blood samples from 50 amphistomes infected as well as 50 non-infected buffaloes from slaughter houses were taken into vacutainer tubes containing ethylene diamine tetraacetic acid for estimation of various hematological parameters by Automatic Analyzer Hema-2062 manufactured by Analytical Technologies Ltd.

**Result:**

There was a significant reduction in the mean hemoglobin, total leukocyte count, total erythrocyte count and packed cell volume and significant increase in the neutrophils count and eosinophil count of infected buffaloes as compared to the non-infected buffaloes respectively.

**Conclusion:**

Amphistomosis is characterized by severe neutrophilia, eosinophilia, and anemia. Anemia of high intensity along with hepatic damage can lead to the death of the animal in severe cases. Alterations in the Hematological parameters can be used as an indicator to diagnose and check the severity of amphistomosis especially in young ones and in subclinical infection.

## Introduction

India has the largest livestock population in the world, which contributes substantially to the national economy, but these ruminants are impressed by multifarious parasites such as nematodes, trematodes and cestodes [1]. Among the helminths, trematode parasites of ruminant livestock have a worldwide distribution and even have zoonotic importance [2]. Among several trematodes affecting ruminants, amphistomes account for huge economic losses [3]. Ruminant amphistomosis is an infection of cattle, buffalo, sheep, goats and other wild ruminants caused by a severe infection with immature amphistomes in the small intestines of immunologically incompetent hosts and considered as a copacetic obstacle in health and production performance of animals throughout the globe [4].

Buffaloes are raised as economically important animals because they are multipurpose; providing milk, meat and good quality hides. They are also used as draft animals (“tractors” in Southeast Asia) in agriculture farms, means of transportation, and their dung act as a good fertilizer [5]. Gastrointestinal parasitic infections of buffaloes are common, which cause considerable global economic losses to the buffalo industry and farming communities as a consequence of mortality in infected animals, reduced weight gain and the condemnation of the affected organs during meat inspection in slaughterhouses [6]. It is distributed ubiquitously throughout the world, but the highest prevalence has been reported in tropical and subtropical regions, particularly in Africa, Asia, Australia, Eastern Europe and Russia. The disease is widely prevalent in India and causes economic loss to the tune of several thousand crores annually [7].

Death due to Paramphistomes is very high and may be as high as 80-90% in domesticated ruminants [8]. Increased production and better health can only be achieved if proper buffalo health management programs including the control of parasitic diseases are planned and effectively implemented [9]. Outbreaks of clinical amphistomosis caused by immature flukes in calves or subclinical infection often pass undiagnosed before the death occurs. This disease is thoroughly understood in various aspects like epidemiology, prevalence rate and its variation based on season, month, age and morphology of causative agent, etc. but on perusal of literature no information on the hematological alterations in buffaloes due to this disease in Gujarat could be investigated.

Keeping this in mind, it was felt necessary to study the alterations in the hematological parameters in buffaloes, to correlate them with subclinical infection of amphistomosis and its impact on the animal health to take appropriate preventive and control measures.

## Materials and Methods

### Ethical approval

Institutional Animal Ethics Committee of Veterinary College, Anand Agriculture University has accorded permission for the collection of blood from the slaughter houses for the study.

### Methods

The study was undertaken for the period of 12 months from March-2013 to February-2014 in Anand and Ahmedabad district of Gujarat, India. Blood samples from 50 amphistomes infected as well as 50 non-infected (apparently healthy) buffaloes were taken during the anti-mortem examination of the animals in the slaughter houses, which were further confirmed during post-mortem examination. Buffaloes for the control group (apparently healthy) were selected on the basis of the anti-mortem examination along with fecal examination, skin scraping examination of the animal followed by detail post-mortem examination of the carcass for diseases, parasites and any abnormalities and the blood was collected to know the correlation between the values obtained from healthy animals and the standard hematological range available which helps in proper estimation of the variation in the parameters during amphistomosis. For blood collection, Animals were bled from the jugular vein into vacutainer tubes containing ethylene diamine tetraacetic acid for estimation of various hematological parameters.

### Parameters studied

Hemoglobin (Hb), total erythrocyte count (TEC), total leucocyte count, differential leucocyte count, packed cell volume (PCV), mean corpuscular Hb (MCH), mean corpuscular volume (MCV) and MCH concentration (MCHC) were analyzed by Automatic Analyzer (Hema-2062 manufactured by Analytical Technologies Ltd, India).

### Statistical analysis

The data generated on hematology were analyzed using Student’s t-test as per the method described by Snedecor and Cochran [10].

## Results

The infected buffaloes in this study showed a highly significant reduction (p<0.01) in the mean Hb, TEC and PCV. There was a significant increase (p<0.05) in the neutrophils count and eosinophil count of infected buffaloes as compared to the non-infected buffaloes. The total leukocyte, monocyte and lymphocyte count decreased non-significantly, and basophil counts increased non-significantly in infected buffaloes (Table-1). There was no significant change in the values of MCV, MCH and MCHC of control (apparently healthy) and infected buffaloes.

**Table-1:** Hematological values of amphistomes infected buffaloes and non–infected buffaloes.

Parameter	Non-infected buffaloes (*n*=50)	Infected buffaloes (*n*=50)
TEC 10^6^/µl	7.607±0.156	5.195±0.086**
TLC 10^6^/µl	8.072±0.055	8.019±0.055
Hb g/dl	9.0753±0.160	8.112±0.069**
PCV %	32.653±0.180	26.317±0.573**
MCV (fl)	42.89±0.240	50.67±0.320
MCH (pg)	11.92±0.194	15.61±0.218
MCHC (g/dl)	27.79±0.128	30.82±0.186
Neutrophils (%)	29.420±0.127	29.909±0.181*
Lymphocytes (%)	56.486±0.241	55.870±0.216
Monocytes (%)	4.588±0.193	4.177±0.347
Eosinophils (%)	4.817±0.142	5.096±0.032*
Basophils (%)	0.240±0.037	0.302±0.043

* p<0.05,

** p<0.01,

TEC=Total erythrocyte count, TLC=Total leukocyte count, Hb=Hemoglobin, PCV=Packed cell volume, MCH=Mean corpuscular hemoglobin, MCV=Mean corpuscular volume, MCHC=Mean corpuscular hemoglobin concentration

## Discussion

Amphistomosis is known as a disease of great concern among parasitic diseases within and outside the country due to various reports of its high prevalence in Pakistan [11], Bangladesh [12], Bihar [13], Andhra Pradesh [14], Udaipur [15], Punjab [16] and New Delhi [3]. Disease, its etiology and its impact on various parameters were thoroughly studied by various scientists. Many scientists reported findings in accordance with the present study *viz*. Panda and Mishra [17] who reported reduction of hemoglobin content and erythrocyte count with eosinophilia in clinical pathological studies of the buffalo calf affected with amphistomosis, Singh *et al*. [18] also observed that there was a significant drop in TEC (p<0.05), Hb (p<0.01), and PCV (p<0.01) in biliary amphistomiasis and significant increase in eosinophilic count (p>0.01) as compared to the healthy animals.

The present study reports anemia and eosinophilia which is in agreement with the earlier reports of Saheb and Hafeez who documented significant decrease (p<0.05) in the hematological values of TEC, PCV and Hb and increased eosinophilic count (p>0.05) in ruminal amphistomiasis as compared to healthy buffaloes [19]. Nath [20] conducted hemato-biochemical changes in cattle with paramphistomiasis and showed a significant decrease in Hb, PCV, TEC and Lymphocyte count and a significant increase in neutrophil and eosinophil count in the diseased animals respectively. Thakur *et al*. [21] studied clinico-hematology on amphistomosis in cattle and revealed eosinophilia. Mavenyengwa *et al*. [22] found significant decrease (p<0.05) in the PCV, Hb and RBC levels occurred from 21 day post-infection, while a significant increase (p<0.05) in the circulating eosinophils occurred between 7 and 21 days post-infection of amphistomosis in cattle. Biswas *et al*. [23] documented hemato-biochemical changes in cattle with naturally acquired paramphistomiasis in which the levels of Hb, TEC and PCV were significantly decreased and increased levels of eosinophil and neutrophil were reported which were in agreement with the present study.

The lower values of TEC, Hb and PCV in infected buffaloes might be due to the loss of blood from severe hemorrhage and ulceration in ruminal mucosa and intestinal tissue due to migration of young immature flukes via the intestine to their predilection site rumen or extensive migration of the young fluke through the hepatic parenchyma and from blood sucking activity of adult fluke. Values of MCV and MCHC in the normal range may also indicate that the loss of blood is of acute nature. With a period of time, the values may decrease further with increase in the infection and the resultant loss of blood. Varma *et al*. [24] studied mineral profile in serum of buffaloes infected with paramphistomosis and showed a significant reduction in iron level that apparently affects the value of Hb causing anemia. Neutrophils are the first line of defense of the host. They are actively amoeboid and phagocytic in nature. The phagocytic action of neutrophils may thus correlate with their increased number in the present study as a check on the infection. The lymphocytes are the second line of cellular defense, and its low number indicates acute infection as there is high demand of lymphocytes in chronic infection. Eosinophilia reported in the present study indicates involvement of parasite in itself.

## Conclusion

In this study, the disease amphistomosis is characterized by severe neutrophilia, eosinophilia and anemia of normocytic normochromic type. Presence of anemia along with the involvement of parasite indicated by eosinophilia can be taken as an indicator to correlate it with subclinical infection of amphistomosis. Anemia of severe intensity can be responsible for mortality in animals especially young ones. Proper treatment by various fluid administration and anti-parasitic drugs may keep a check on mortalities due to subclinical infection that is hard to diagnose till severity rises.

## Authors’ Contribution

This study is the major component of the work toward the M.V.Sc thesis of the first author VDC, under the guidance of PVP and JJH. SSP and DVP helped in sample collection from various abattoirs. SP and VJ helped in blood profiling, organized and thoroughly revised the manuscript. All authors have read and approved the final version of the manuscript.
